# Bridge Builders and Boundary Spanners: Intersectoral, Interorganizational, and Interprofessional Education

**DOI:** 10.1093/geroni/igab046.1678

**Published:** 2021-12-17

**Authors:** Phillip Clark

**Affiliations:** University of Rhode Island, Kingston, Rhode Island, United States

## Abstract

The needs of individuals aging with intellectual and developmental disabilities (IDD) and their families do not fall neatly within defined policies, programs, and professions. They comprise complex challenges based on medical, psychological, social, environmental, economic and familial dimensions. These needs pose a challenge for providers in developing solutions at three levels: (1) different policies and programs create barriers based on different funding sources, eligibility requirements, and administrative restrictions; (2) clinical and community-based programs embody the gap between healthcare and human service providers; and (3) different professions are trained in their own methods of assessment and care plan development that impede the design of integrated approaches to defining and solving problems. This paper proposes an intersectoral, interorganizational, and interprofessional framework for addressing these problems based on networking and collaborative practice principles embodying bridge-building, boundary-spanning, and team-working as a basis for provider education. Implications for expanded education in this field are explored.

